# Generation of germline ablated male pigs by CRISPR/Cas9 editing of the *NANOS2* gene

**DOI:** 10.1038/srep40176

**Published:** 2017-01-10

**Authors:** Ki-Eun Park, Amy V. Kaucher, Anne Powell, Muhammad Salman Waqas, Shelley E.S. Sandmaier, Melissa J. Oatley, Chi-Hun Park, Ahmed Tibary, David M. Donovan, Le Ann Blomberg, Simon G. Lillico, C. Bruce A. Whitelaw, Alan Mileham, Bhanu P. Telugu, Jon M. Oatley

**Affiliations:** 1 Department of Animal and Avian Sciences, University of Maryland, College Park, MD 20742, USA; 2 Animal Bioscience and Biotechnology Laboratory, USDA, ARS, Beltsville, MD 20705, USA; 3 Renovate Biosciences Inc, Riesterstown, MD, USA; 4 Center for Reproductive Biology, College of Veterinary Medicine, Washington State University, Pullman, WA 99164, USA; 5 Roslin Institute, University of Edinburgh, Edinburgh, Scotland; 6 Genus PIC, De Forest, WI, USA

## Abstract

Genome editing tools have revolutionized the generation of genetically modified animals including livestock. In particular, the domestic pig is a proven model of human physiology and an agriculturally important species. In this study, we utilized the CRISPR/Cas9 system to edit the *NANOS2* gene in pig embryos to generate offspring with mono-allelic and bi-allelic mutations. We found that *NANOS2* knockout pigs phenocopy knockout mice with male specific germline ablation but other aspects of testicular development are normal. Moreover, male pigs with one intact *NANOS2* allele and female knockout pigs are fertile. From an agriculture perspective, *NANOS2* knockout male pigs are expected to serve as an ideal surrogate for transplantation of donor spermatogonial stem cells to expand the availability of gametes from genetically desirable sires.

Spermatogenesis is a robust and dynamic developmental process that occurs in seminiferous tubules of testes and generates millions of sperm each day in males from puberty through advanced age. The undifferentiated spermatogonia provide the foundation for spermatogenesis^1^,^2^. Mitotic proliferation by transit amplifying progenitor spermatogonia builds a pool that will periodically transition to a differentiating state, at which point the cells are committed to terminal differentiation as sperm. A rare subset of undifferentiated spermatogonia possess self-renewal and regenerative capacity to serve as a spermatogonial stem cell (SSC) pool from which a new cohort of progenitor spermatogonia will arise.

Aside from a role in sustaining steady-state spermatogenesis, SSCs are also capable of regenerating the spermatogenic lineage following cytotoxic damage that eliminates the progenitor population, or following transplantation into the seminiferous tubules of a germ cell ablated recipient male^3^,^4^. Methodology for transplantation of SSCs that results in reestablishment of fertility is developed for rodents but, extension to higher order mammals has not been reported. For livestock, SSC transplantation has the potential for use as a tool to expand the availability of gametes from desirable (elite) sires, and thereby dramatically influence production efficiency, quality and other production traits in a population^5^,^6^. A key aspect for successful SSC transplantation is the requirement for recipient males with intact seminiferous tubules and somatic support cell populations, but lacking SSCs and other germ cells. The current available options to achieve this outcome are not ideal. For example, treating with chemotoxic drugs (busulfan)^4^,^7^, either fails to completely eliminate the endogenous germline or has undesirable side effects on animal health. Likewise, local irradiation can cause damage to somatic cells. These drawbacks necessitate a need for an alternative approach for livestock species that results in targeted ablation of SSCs but spares testicular somatic cells.

The precursors of SSCs are prospermatogonia that are derived from primordial germ cells (PGCs) during fetal development^8^. PGCs are bi-potential during early embryogenesis but at the time of sex determination transition into prospermatogonia in XY fetuses and oocytes in XX fetuses^9^. In all mammals studied to date, the SSC pool then develops from prospermatogonia during a defined window of time in neonatal life. In mice, impaired formation or survival of prospermatogonia leads to a loss of germline prior to establishment of the spermatogenic lineage but seminiferous tubules remain intact^10^,^11^. Thus, elimination of prospermatogonia could be a viable strategy to develop germline ablated males that would serve as potential recipients for exogenous SSC transplantation from a donor male.

Beyond the generation of recipient models for SSC transplantation, understanding the mechanisms influencing prospermatogonial formation and survival, as well as SSC formation, could help inform cases of infertility in men. Non obstructive azoospermia (NOA) is a condition that describes the complete lack of sperm in the ejaculate. In many NOA cases, the cause is lack of germline, a condition referred to as “Sertoli-cell-only” syndrome^12^. Because the testes seem to develop normally and testosterone is produced, diagnosis of NOA and Sertoli-cell-only syndrome are almost always made after puberty. It is possible that many instances of male infertility are caused by lack of prospermatogonia in early development.

The NANOS family of RNA binding proteins are known to play an essential role in the development of germline for all organisms studied to date. In Drosophila, the single *nanos (nos*) gene encodes for a protein that is required for development of both male and female germline^13^. Interestingly, mice possess three *Nanos* paralogs, each with a different role in germline development. Deficiency of *Nanos1* has no known effect on growth and development of any systems including the germline^14^. In contrast, *Nanos3* deficiency leads to male and female sterility due to impaired survival of PGCs prior to sex determination^10^. Importantly, *Nanos2* knockout male mice are sterile due to apoptosis of prospermatogonia shortly after birth, however, homozygous knockout females, as well as heterozygous knockout males and females possess normal germline and are fertile^10^,^11^. Therefore, the choice of *NANOS2* for targeting in livestock has two advantages: (1) homozygous *NANOS2* knockout males will be deficient in SSCs and will serve as ideal recipients for transplantation; and (2) heterozygous knockout males when bred to homozygous knockout females (both fertile) will efficiently propagate the line and generate *NANOS2* null animals at high frequency.

In this study, CRISPR/Cas9 technology was utilized to produce inactivating mutations in the *NANOS2* gene of domestic pigs. Outcomes revealed that male pigs with bi-allelic frameshift mutations (i.e. knockouts) resulted in ablation of germline but with morphologically intact seminiferous tubules. Also, males with heterozygous frameshift mutations and females with homozygous and heterozygous frameshift mutations are fertile. These observations confirm that genetic modification of *NANOS2* in pigs phenocopy mutations in mice.

## Results

### Generation of *NANOS2* mutant pigs

**Figure 1:**
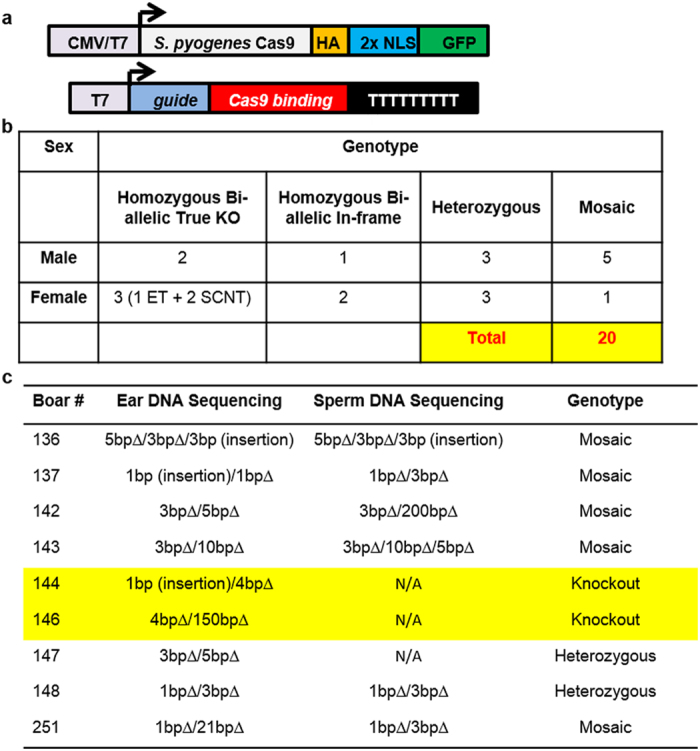
Generation of *NANOS2* gene edited pigs. (**a**) Schematics of Cas9:GFP expression vectors containing an HA tag, nuclear localization signal (NLS) and CMV promoter for mammalian *in vivo* expression (top) or T7 promoter for *in vitro* transcription (bottom). (**b**) Distribution of genotypes for 20 genome edited piglets of 3 litters produced from microinjected *in vivo* derived embryos (n = 18) and 2 somatic cell nuclear transfer (SCNT) piglets. (**c**) Genotype of 9 mono- and bi-allelically edited boars from ear biopsy and semen DNA samples. Two of the knockout boars that had ORF disrupting mutations and no spermatogenesis are highlighted in yellow. The alleles were determined by PCR amplification and sub-cloning of a 500 bp amplified fragment from each piglet (flanking the target site of *NANOS2* genomic site) into PCR2.1 plasmid and sequencing of 10 bacterial clones from each piglet. Two of the knockout boars (#144 and #146) that had ORF disrupting mutations had no spermatogenesis, and hence no genotyping from spermatozoa could be performed. Boar #147 was euthanized and no further genotyping was performed.

In pigs, *NANOS2* is a single exonic gene with an open reading frame (ORF) of 417 bp. A candidate CRISPR/Cas9 single guide RNA (sgRNA) was designed to target 48 to 70 bp proximal to the translational start site (AUG) (Supplementary Figure S1). To produce pigs with mutations in the *NANOS2* gene, donor animals were estrous synchronized, artificially inseminated, and *in vivo* fertilized zygotes and unfertilized oocytes were surgically recovered. The *in vivo* fertilized zygotes were microinjected with a mixture of sgRNAs and a SpCas9 RNA expression construct (Fig. 1a), and 30 zygotes were transferred into each of the synchronized recipient gilts. The remaining unfertilized oocytes were *in vitro* fertilized, injected with the CRISPR cocktail and all 54 zygotes surgically transferred. Three out of four recipients from the embryo transfers were confirmed pregnant that resulted in the birth of 12 males and 6 females, total 18 live piglets (Fig. 1b and Supplementary Figure S2). Genotyping of ear notch biopsies identified an array of mutations with all piglets showing either mono- or bi-allelic modifications and no wildtype animals (Fig. 1c and Supplementary Figure 2). Among the male offspring, piglets #137, 144, 146 and 251 showed bi-allelic frameshifting mutations, piglet #136 had three confirmed edited alleles (mosaic), whereas, piglets #142, 143, 147, and 148 contained at least one 3 bp in-frame deletion resulting in the loss of valine (at position 19). We hypothesized that 3 bp in-frame deletions would be functional, and piglets #142, 143, 147 and 148 were classified as heterozygous null, and piglets #137, 144, 146 and 251 with out-of-frame bi-allelic modifications as homozygous knockouts. Three of the littermates (2 heterozygous and 1 homozygous null) were euthanized prior to or around the time of weaning and were, therefore not included in the study.

### Growth and development of *NANOS2* mutant male pigs

**Figure 2:**
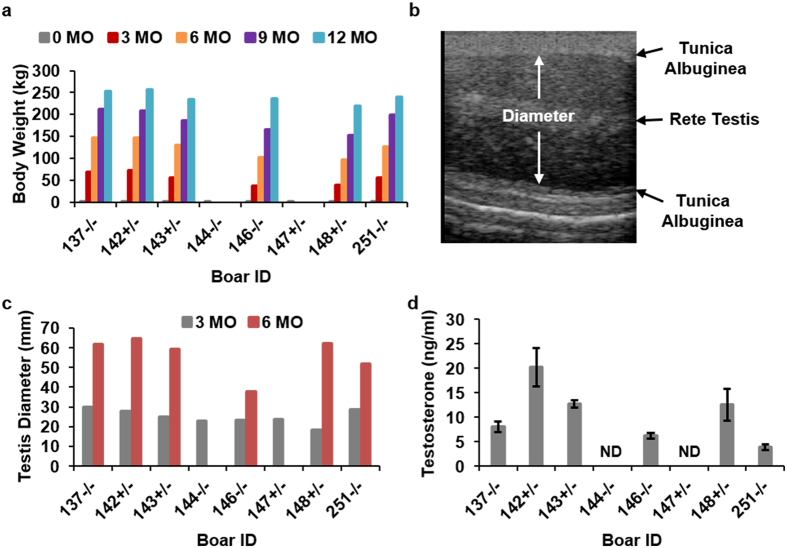
Growth and development of *NANOS2* gene edited male pigs. (**a**) Body weight of individual *NANOS2* heterozygous edited (#142, 143, 147, 148) and homozygous edited (#137, 144, 146, 251) male pigs at periodic points in development from birth (0 months, MO) through adulthood (12 MO). Note that data are not presented for pigs #144 and 147 at the age points of 6-12 MO because they were euthanized around 7 MO of age for health reasons. (**b**) Representative ultrasound image of a testis from *NANOS2* gene edited pigs. Image is from bi-allelic *NANOS2* edited pig #137. (**c**) Testis diameter measurements from ultrasound imaging for individual *NANOS2* mono-allelic edited (#142, 143, 147, 148) and bi-allelic edited (#137, 144, 146, 251) male pigs during prepubertal development (3 MO) and at puberty (6 MO). Note that data are not presented for pigs #144 and 147 at the 6 MO age point because of deteriorating health. (**d**) Serum testosterone concentration for individual *NANOS2* mono-allelic edited (#142, 143, 148) and bi-allelic edited (#137, 146, 251) male pigs at 7 months of age. Data are presented as mean ± STDV for all pigs of each genotype. Note that in the figure ND stands for “Not determined” as pigs #144 and 147 were euthanized prior to the serial blood sampling.

We reasoned that if *NANOS2* plays a role in the development and function of cell lineages other than the male germline in pigs, overall growth and development would be affected. To assess this, we measured the weight of male animals at periodic intervals from birth through adulthood (Fig. 2a). At birth, the average weight of homozygous knockout *NANOS2* male pigs was 1.3 ± 0.2 kg (mean ± STDV and n = 4) which was not different (P = 0.19) compared to the average weight of heterozygous knockout males, 1.9 ± 0.3 kg (n = 4). Likewise, there was no difference (P = 0.42) in body weight between the homozygous and heterozygous groups at weaning (8.0 ± 1.1 vs. 9.1 ± 0.2, respectively; n = 4 of each genotype), or at 3, 6, 9, or 12 months of age (Fig. 2a). In addition, no gross differences in the appearance or behavior of animals were observed. Note that homozygous knockout pig #144 and heterozygous pig #147 became ill around 7 months of age and had to be euthanized, thus these animals are not included in the full analysis. Boar #144 developed lameness in the right forelimb that limited standing and movement and boar #147 developed an esophageal blockage that impaired his ability to eat. To assess testis development, we used ultrasound imaging to measure the diameter of each testis at periodic age points (Fig. 2b and c). At 3 months of age, the average paired testis diameter for homozygous null edited pigs was 26.1 ± 1.3 mm (mean ± SEM and n = 4 of each genotype) which was not different (P = 0.24) from the 23.7 ± 0.8 mm diameter for heterozygous animals (n = 4). At 8 months of age, the average paired testis diameter for homozygous null edited boars #137 and 251 was 61.9 ± 2.6 mm (mean ± STDV) and 51.9 ± 6.0 mm (mean ± STDV), respectively, which was similar to the 62.1 ± 4.5 mm average paired testis diameter of the heterozygous edited group. In contrast, the average paired testis diameter for homozygous edited boar #146 was reduced by ~40% to 37.5 ± 1.8 (mean ± STDV) compared to the heterozygous group. A phenotype of reduced testis size could be caused by disruption of the hypothalamic-pituitary-gonadal (HPG) axis. To assess this possibility, we measured serum testosterone concentration in serial samples (4 time points at 15 min intervals) from heterozygous and homozygous edited animals at 6 months of age (Fig. 2d). Although the mean value for each boar varied, all were within the normal range for an adult male^15^. Taken together, these findings suggested that loss of *NANOS2* expression does not alter normal growth and development in male pigs. However, overall development of testes is impaired, at least in some knockout animals, possibly due to disrupted establishment of the germline. The disparity in testis diameter phenotype among the homozygous edited boars suggested variability in *NANOS2* loss-of-function or mosaicism of the edited alleles in some animals.

### Germline ablation of *NANOS2* male knockout pigs

**Figure 3:**
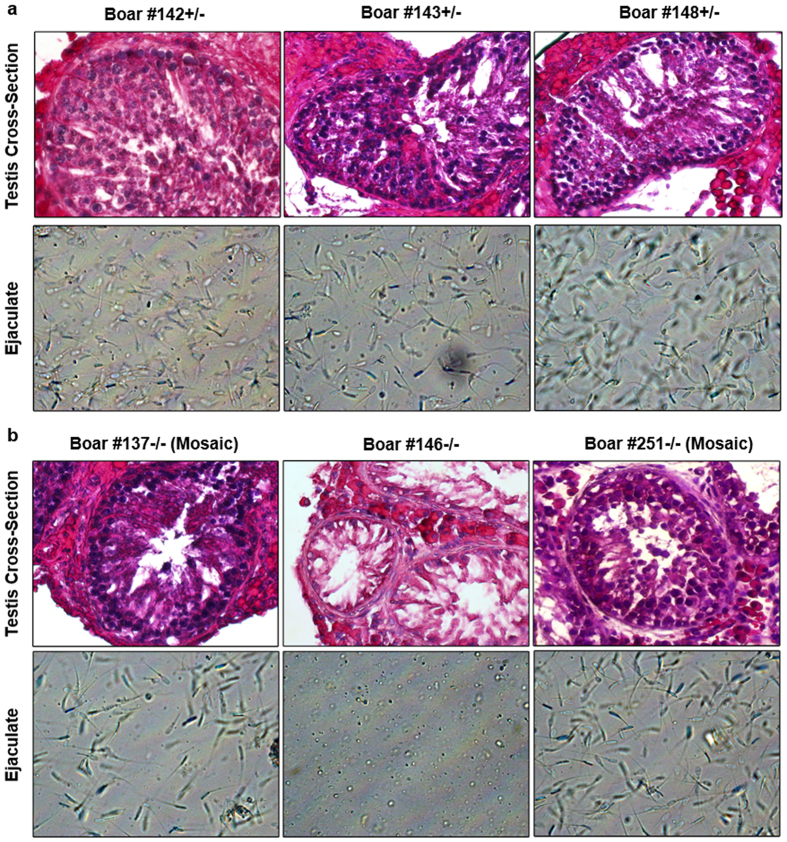
Testicular phenotype of *NANOS2* gene edited male pigs. (**a** and **b**) Representative images of cross-sections from testicular parenchyma (upper panels) and ejaculates (lower panels) from *NANOS2* mono-allelic (**a**) and bi-allelic (**b**) edited pigs at adulthood (6–8 months of age). Note that the cross-section of testicular tissue from bi-allelic knockout pig #146 lacks germline and the ejaculate is devoid of sperm.

To assess the testis phenotype of *NANOS2* mutant pigs further, we collected testicular biopsies from both homozygous knockout and heterozygous animals at the pubertal age of 6–8 months. Evaluation of cross-sections revealed the presence of intact seminiferous tubules for both genotypes and germ cells were clearly evident in tubules of all heterozygous null males (Fig. 3a), as well as homozygous edited boars #137 and 251 (Fig. 3b). However, no germ cells were apparent in tubules of homozygous edited boars #144 (Supplementary Figure S3) and 146 (Fig. 3b). In adulthood, semen samples were collected from the boars using a dummy apparatus. Samples from heterozygous edited animals contained numerous sperm with a progressive motility and normal morphology of >90% (Fig. 3a). Similarly, sperm were present in the ejaculates of homozygous edited boars #137 and 251 (Fig. 3b). In contrast, no sperm were detectable in samples from homozygous edited boar #146 (Fig. 3b). Note that boar #144 became ill and semen collection was not possible. Taken together, these results led us to speculate that bi-allelic edited boars #137 and 251 might be mosaics in which at least one functional *NANOS2* allele was intact in the germline. Indeed, genotyping of semen from boars #251 and 137 revealed at least one non-inactivating *NANOS2* allele (Fig. 1c). Thus, bi-allelically edited boars #144 and 146 were true *NANOS2* knockouts; whereas, bi-allelically edited boars #137 and 251 were mosaics with a functional *NANOS2* allele in germ cells. Collectively, these findings demonstrate that the frameshifting true knockout of *NANOS2* results in germline specific ablation in males (boars #144 and 146), which is conserved from mouse to pig.

### Fertility of female NANOS2 mutant pigs

**Figure 4:**
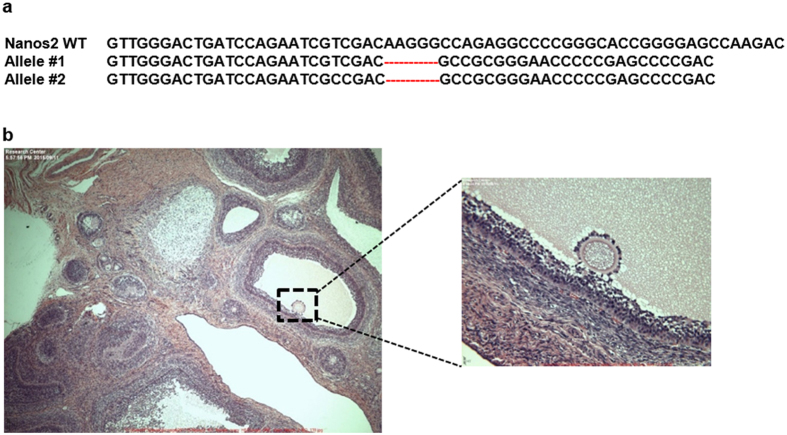
*NANOS2* is dispensable for germline development and fertility in female pigs. (**a**) Genotype of cloned *NANOS2* null gilt showing bi-allelic 11 bp frameshifting mutations. (**b**) Histological analysis of ovary from a 7 month old null gilt showing active folliculogenesis (left). Magnified view of one of the follicles showing a growing oocyte (right).

From the embryo transfers, we identified 6 female piglets, none of which possessed homozygous out-of-frame mutations (Supplementary Table S1). We therefore nucleofected somatic cells with CRIPSR/Cas9 GFP plasmid and a sgRNA expression vector targeting the *NANOS2* locus. One of the clonal lines that was bi-allelic knockout for *NANOS2* was used as a donor for somatic cell nuclear transfer (SCNT), which yielded true knockout female piglets (Fig. 4a). Note that nuclear transfer is performed with one starting cell of a known genotype as the nuclear donor. Therefore, unlike the animals derived from zygote injections, mosaicism or the presence of an unaccounted 3^rd^ allele is not possible in SCNT -derived animals. Beginning at 8 months of age, the knockout females exhibited normal estrous cyclicity (19–21 days) and evaluation of cross-sections from the ovaries of one piglet (euthanized because of musculoskeletal issues) revealed intact follicles and normal folliculogenesis (Fig. 4b). Breeding of a clonal *NANOS2* knockout littermate resulted in a successful pregnancy (confirmed by ultrasound at 4 weeks). Lastly, we utilized sperm from the mosaic heterozygous knockout boar #142 to breed NANOS2 mutant sows #145 and #149 (heterozygous knockouts), and boar #143 was bred to sow #140 (heterozygous knockout); all of which resulted in pregnancies and produced litters (Supplementary Table S2). Taken together, these findings demonstrate that the germline is intact in females that are deficient for *NANOS2*.

## Discussion

The domestic pig is both a model for studying mammalian physiology and an important species for agricultural applications. The capacity to manipulate the genome of domestic pigs allows for producing higher order mammalian research models to further define the role of specific allelic variants in both physiological processes, as well as the development and progression of disease. Furthermore, domestic pigs are a major food source worldwide and applying genome modification can produce lines with enhanced efficiency for growth, disease resistance, and biopharmaceutical properties. However, the domestic pig has not traditionally been amenable to genetic manipulation due in part to long generation interval and lack of tools that facilitate genetic modification. The advent of site-specific nucleases including zinc finger nucleases, TALENS and more specifically CRISPR/Cas9 that induce a double stranded break at the target site and generate knockouts and knockins have accelerated genetic engineering and generation of pig models^16^,^17^,^18^,^19^.

In this study, we have shown that direct injection of CRISPR reagents into the cytoplasm of *in vivo* and *in vitro* fertilized zygotes resulted in 18 piglets from three litters, with all piglets showing modifications in one (1 piglet) or both alleles (17 piglets). The identification of edits in a mono- or bi-allelic fashion in all piglets (and no wildtype offspring) points to the high efficiency of the CRISPR/Cas9 editing approach. That said, one third of the bi-allelically edited piglets (6 piglets) were found to be mosaic with 3 different alleles in the animals. Notably, 4/5 mosaic males (#136, 137, 142, and 143) were from injection of *in vivo* fertilized zygotes, compared to only one mosaic animal from injection of *in vitro* fertilized zygotes. The high incidence of mosaicism could be a result of injection into *in vivo* zygotes that are close to their first cell division resulting in independent editing events in two cells. In pigs, ovulations and consequently fertilization occurs over a broader window of time. This feature, coupled with harvest of embryos at different times could result in developmental asynchrony, with some embryos being more advanced than others in development. Conversely, harvest of oocytes that were *in vitro* fertilized are expected to be more synchronous. Another possibility is the persistence of CRISPR reagents and Cas9 protein past the first cell division, again resulting in differential modification and repair in the two cells. Relevant to this, the authors have noted that the use of *in vivo* matured oocytes that mount a better block to polyspermy, followed by *in vitro* fertilization, and injection of CRISPR ribonucleoproteins (instead of *Cas9* mRNA) has no incidence of mosaicism in resulting offspring (Telugu lab; unpublished data).

The role of NANOS proteins in germ cell development is well understood for model organism but undefined for higher order mammals. In the current study, we discovered that inactivation of *NANOS2* in the domestic pig phenocopies the mutations in the mouse. Prior to puberty, no difference in testis development, based on paired diameter measurements was observed between homozygous (#144 and #146) and heterozygous *NANOS2* littermates. At puberty, the size of the testes had dramatically increased for pigs with *NANOS2* sufficiency but limited growth occurred from the knockout male. Importantly, an abundance of sperm were visible in the ejaculates of pigs with functional *NANOS2* alleles but not evident in the ejaculate of knockout pig #146 and evaluation of cross-sections of testicular tissue from knockout boars #144 and #146 confirmed germline ablation but intact seminiferous tubules. In addition, serum testosterone concentration was measured to be in the normal range for knockout pig #146 and the heterozygous edited and mosaic littermates, thus demonstrating functional testicular interstitial tissue and an intact HPG axis. Lastly, female *NANOS2* knockout pigs are fertile and therefore possess an intact germline. Although comparison to wild-type littermates would have provided a more well-rounded perspective of the findings, the high efficiency of the CRISPR/Cas9 system to mutate at least one allele in each pig negated this possibility. Regardless, taken together, these findings demonstrate a male specific role for *NANOS2* in germline maintenance.

Although the role of *NANOS2* in male germline maintenance for model organisms (and now a higher order mammal) is demonstrated, the mechanisms of its actions and mode of germ cell loss is still undefined. The *NANOS2* knockout pig is expected to serve as a valuable bridge model for filling these gaps in knowledge. Polymorphisms in the *NANOS* genes are associated with NOA in men^20^,^21^. Considering that the defining criterion of the condition is absence of sperm in the ejaculate, which is typically caused by germline ablation, deficiency in *NANOS2* could be the likely factor. Because germline and testis development in the pig is similar to humans, studying how germ cell loss occurs in *NANOS2* knockout pigs may yield valuable information for devising therapeutic/diagnostic approaches to prevent loss in humans.

Another important aspect of the *NANOS2* knockout male phenotype is that seminiferous tubules and testicular interstitial tissue function appear to be intact. Thus, these animals may serve as ideal recipients for transfer of male germline via SSC transplantation. When successful, donor-derived spermatogenesis would occur in the *NANOS2* knockout recipient tubules without mixing the endogenous germ cells. Thus, 100% of offspring sired from breeding of the recipients via natural service or *in vitro* fertilization would possess the donor male’s haplotype. This paradigm would be extremely valuable for production animal agriculture as both a means to expand the availability of gametes and preserve the germline of genetically desirable sires. Importantly, the fact that male pigs with one inactive *NANOS2* allele and female pigs with two inactive *NANOS2* alleles are fertile allows for efficient expansion of the line and generation of sufficient knockout males for experimentation. As a necessary first step, we setup crosses of the heterozygous males and females and three litters have been generated (Supplementary Table S2). Additionally, SCNT was performed with fibroblasts from homozygous knockout boar #146 to generate additional clones (n = 6; Supplementary Figure S4). Now that the indispensable role of *NANOS2* in male germline is established in pigs, the goal of future studies will be to test the suitability of using *NANOS2* knockout males as surrogates for SSC transplantation.

## Methods

### Animals

All animal procedures and experimentation were approved by the Institutional Animal Care and Use Committee (IACUC) of Washington State University and the USDA Animal Research Service (Beltsville) in accordance with the Guide for the Care and Use of Agricultural Animals in Research 3^rd^ edition. Domestic PIC pigs of the large white (LW) and Landrace (LR) breeds were crossbred to produce F1s that were utilized in this study.

### Generation of NANOS2 edited animals via embryo injections

A candidate chimeric sgRNA targeting Exon-1 of *NANOS2* was designed based on the software developed by Dr. Feng Zhang at MIT (http://crispr.mit.edu/). The sequence of the Guide RNA used is: GATCAGTCCCAACACCACCTGG (20 nucleotide guide sequence and the PAM motif (underlined)). A second guide RNA: GAATCGTCGACAAGGGCCAGAGG, was used in the nuclear transfer experiment. The candidate CRISPR sgRNAs, in combination with Cas9:GFP mRNA, were *in vitro* transcribed using a T7 mMessage Machine kit (Ambion), cleaned with a Megaclear Kit (Ambion) and microinjected into the cytoplasm of *in vivo* or *in vitro* fertilized porcine one-cell embryos. To prepare embryo donors, nine pubertal gilts were subjected to estrus synchronization by treatment with the progesterone analog Regumate (24–36 mg/animal) for 14 days. Females in standing heat were then artificially inseminated (AI) with commercial boar semen provided by PIC. The *in vivo* derived embryos were recovered surgically 24 hours after AI by retrograde flushing with sterile PVA TL-Hepes medium from the oviduct. Fertilized embryos were then injected with Cas9:GFP mRNA and sgRNA targeting *NANOS2*, and cultured in PZM3 medium^22^. Embryos were transferred surgically into the oviducts of estrus synchronized surrogate females five hours after injection (n = 3 recipients receiving 30 embryos each). Non-fertilized oocytes were fertilized *in vitro*. The oocytes were placed in 100 μl of a modified Tris-buffered medium (mTBM) and fertilized according to established protocol^23^ using fresh extended boar semen. One ml of extended semen was mixed with Dulbecco’s Phosphate Buffered Saline (DPBS) containing 1 mg/ml BSA to a final volume of 10 ml and centrifuged at 1000 × g, 25 °C for four minutes, and spermatozoa were washed in DPBS three times. After the final wash, spermatozoa were re-suspended in mTBM medium and added to oocytes at a final concentration of 5 × 10^5^ spermatozoa/ml, and co-incubated for 5 h at 38.5 °C and 5% CO2. Presumptive zygotes were microinjected five hours later and transferred to a surrogate female after 18 hours (n = 1 recipient receiving 54 embryos). Pregnancies were confirmed by lack of return to estrus (21 days) and ultrasound at 28 days post embryo transfer. For SCNT, the Cas9:GFP plasmid, in combination with a hU6 promoter driven sgRNA vector, were co-transfected into porcine somatic cells and sorted as single cells into a 96-well plate. Clonal lines were obtained by culture for two weeks, genotyped, and confirmed lines were used for cloning as a service by MOFA Global (WI, USA).

### *NANOS2* genotyping analysis

Tissue biopsies (ear notch) from fetuses and offspring were digested in a tissue lysis buffer (50 mM Tris pH 8.0, 0.1 M NaCl, 20 mM EDTA, 1% SDS, 50 μg/ml RNase A, 100 μg/ml proteinase K) overnight at 65 °C. Next, genomic DNA was extracted using phenol-chloroform, and recovered by resuspension in 100 μl of 10 mM Tris- HCl, pH 7.4 buffer following ethanol precipitation. Purified genomic DNA was amplified using PCR, cloned into PCR2.1 vectors (Life Technologies) and transformed into *Ε. coli* DH5-α maximum competent cells (Life Technologies). Five to ten colonies were picked, cultured, plasmid DNA extracted and sequenced (Macrogen). Sequences were aligned by Bio-Edit software for comparison with wild-type alleles. Primers for genotyping are provided in Supplementary Data 4.

### Testicular ultrasound

Testes of boars at the immature and adult stages of development were imaged using a Sonoscape ultrasound machine and static images were captured to measure the diameter of testes.

### Testicular biopsy and cross-sectional analysis

To assess whether seminiferous tubules were intact and the germline was present in *NANOS2* mutant male pigs, biopsies of parenchyma were collected for cross-sectioning. Briefly, boars were placed under general anesthesia, a small incision made in the scrotum and an 18 gauge biopsy punch was inserted into the testicular parenchyma and ~100 mg of tissue was removed. The tissue was then fixed 2–3 hours in Bouin’s solution followed by washing in 70% ethanol and processing for paraffin embedding. Cross-sections of 5 μm thickness were adhered to glass slides, deparrafanized, and then stained with hematoxylin and eosin.

### Blood sampling and testosterone measurements

To evaluate functionality of the hypothalamic-pituitary gonadal (HPG) axis in *NANOS2* mutant pigs, serum testosterone concentrations were measured using LC-MS. Briefly, blood samples were collected every 15 minutes for 1 hour because testosterone is secreted in a pulsatile manner. Samples were then centrifuged to separate serum and plasma and the serum stored at −20 °C before shipment to the Endocrine Technologies Support Core (ETSC) at the Oregon National Primate Research Center (ONPRC) for measurement of testosterone by LC-MS analysis (see supplemental information for detailed procedure).

### Semen collection and analysis

Assessment of sperm production by *NANOS2* mutant boars was conducted by collecting semen samples. Briefly, boars were trained at a young peri-pubertal age on a dummy apparatus (MOFA) for manual semen collection. Samples were diluted in commercial extender solution (MOFA) and analyzed by light microscopy.

### *NANOS2* knockout gilt estrus detection and insemination

The *NANOS2* females were heat checked daily and estrus activity was first noted at five months of age with regular 21 day cycles. Estrus in the gilts was detected by noting visible signs of estrus behavior including increased physical activity, phonation, pointed ears, and lordosis (arching of the back in response to physical pressure) in the presence of teaser boar. The gilts were bred to heterozygous *NANOS2* boars (10–16 months) or PIC semen at 10 months and confirmed pregnant by ultrasound 28 days later.

### Data presentation and statistical analysis

All quantitative data are presented as either the mean ± SEM or mean ± STDV for at least 3 different animals of each genotype or as values for each individual animal. Differences between means were examined statistically using a two-tailed t-test and a P-value of <0.05 was considered significant.

## Additional Information

**How to cite this article**: Park, K.-E. *et al*. Generation of germline ablated male pigs by CRISPR/Cas9 editing of the *NANOS2* gene. *Sci. Rep.* **7**, 40176; doi: 10.1038/srep40176 (2017).

**Publisher's note:** Springer Nature remains neutral with regard to jurisdictional claims in published maps and institutional affiliations.

## Supplementary Material

Supplementary Information
