# Supplementary material for: Generation of germline ablated male pigs by CRISPR/Cas9 editing of the NANOS2 gene
Source: Sci Rep. 2017 Jan 10;7:40176. doi: 10.1038/srep40176 (PMC5223215; doi:10.1038/srep40176)
Supplement: Supplementary Information [file srep40176-s1.pdf]

Title: Generation of germline ablated male pigs by CRISPR/Cas9 editing of the *NANOS2* gene

Authors: Ki-Eun Park[1,2,3*], Amy V. Kaucher[4*], Anne Powell[2], Muhamad Salman Waqas[4], Shelley E.S. Sandmaier[1,2], Melissa J. Oatley[4], Chi-Hun Park[1,2], Ahmed Tibary[4], David M. Donovan[2], Le Ann Blomberg[2], Simon G. Lillico[5], C. Bruce A. Whitelaw[5], Alan Mileham[6], Bhanu Telugu[1,2,3], and Jon M. Oatley[4]

Affiliations:

[1]Department of Animal and Avian Sciences, University of Maryland, College Park, MD 20742

[2]Animal Bioscience and Biotechnology Laboratory, USDA, ARS, Beltsville, MD 20705

[3] Renovate Biosciences Inc., Riesterstown, MD

[4]Center for Reproductive Biology, College of Veterinary Medicine, Washington State University, Pullman, WA 99164

[5]Roslin Institute, University of Edinburgh, Edinburgh, Scotland

[6]Genus PIC, De Forest, WI

*Contributed equally

Correspondence: joatley@vetmed.wsu.edu or btelugu@umd.edu

Supplementary Methods

Total serum testosterone levels were measured by the Endocrine Technologies Support Core (ETSC) at the Oregon National Primate Research Center (ONPRC, Beaverton, OR) using ultra-high performance liquid chromatography-heated electrospray ionization-tandem triple quadrupole mass spectrometry (LC-MS/MS) on a Shimadzu Nexera-LCMS-8050 instrument (Kyoto, Japan). For sample preparation, 100 μl of serum were mixed with 100 ul ultrapure water (Milli-Q, EMD Millipore, Billerica, MA) containing 0.8 ng/ml testosterone-C3 isotopic standard (Cerilliant, Round Rock, TX) and added to a 400 μl SLE+ extraction plate (Biotage, Charlotte, NC). Testosterone was eluted with 2 x 900 μl dichloromethane (Sigma, St. Louis, MO), dried with forced air and reconstituted in 50 μl of 25% (v:v) methanol:ultrapure water. Quality controls (QCs) were prepared by spiking testosterone standard into normal rhesus macaque serum, yielding concentrations of 4 ng/ml and 6 ng/ml. QCs were subjected to the same SLE+ extraction procedure with four replicates in each assay. For calibration curves, charcoal-stripped human serum (Golden West Biologicals) was spiked with testosterone standard (Cerilliant) in methanol and diluted serially to final concentrations between 0.009 and 10 ng/ml in a 12-point curve. The spiked standards were then subjected to the SLE+ extraction procedure. After the reconstitution step, samples were subjected to LC-MS/MS analysis. Using a Shimadzu SIL-30CAMP autosampler, 25 μl of each sample were injected onto a Raptor 2.7 μm Biphenyl 50 mm X 2.1 mm column (Restek, Bellefonte, PA). Mobile phase consisted of 0.2 mM ammonium fluoride (Sigma) in water (A), and methanol (B) with a flow rate of 0.25 ml/min. Using a Shimadzu Nexera LC-30AD system (LC), gradient elution started at 70% B and increased to 100% B over 4 minutes. After chromatography, 4.30 minutes were spent re-equilibrating the column back to 70% B for a total of 8.30 minutes/sample. Testosterone had a retention time of 3.45 min. Testosterone was detected in positive ion mode with multiple reaction monitoring (MRM) using a Shimadzu LCMS-8050 tandem triple-quadrupole MS with heated electrospray ionization (ESI). The MRM transitions used were: testosterone, 289.10→97.20 (quant), 289.10→109.25 (qual), *m/z*; testosterone-C3, 292.05→100.40 (quant), 292.05→112.00 (qual), *m/z*. The interface temperature was 300°C, the desolvation line temperature was 150°C, and the heat block temperature was 500°C. The dynamic range for the testosterone standard curve was 0.009 to 10 ng/ml. Data processing and analysis was performed using LabSolutions Software, V5.72 (Shimadzu, Kyoto, Japan). Intra-assay variation was 10%. Because all samples were analyzed using one assay, no specific inter-assay variation was calculated for this experiment. Overall inter-assay variation for this assay in the ETSC is less than 12%.

Supplementary Figure S1. CRISPR guide RNA sequence used in the embryo injection studies is identified in the *NANOS2* ORF (Note: Guide is in reverse orientation, underlined and PAM motif highlighted in blue). A second guide used for SCNT experiment is also shown (in forward orientation, underlined, and PAM motif highlighted in grey).

```
  1 ATGCAGCTGC CACCCTTTGA CATGTGGAAG GACTACTTCA ACCTGAGCCA
    M  Q  L  P   P  F  D  M  W  K   D  Y  F  N   L  S  Q

 51 GGTGGTGTTG GGACTGATCC AGAATCGTCG ACAAGGGCCA GAGGCCCCGG
     V  V  L   G  L  I  Q   N  R  R   Q  G  P   E  A  P  G

101 GCACCGGGGA GCCAAGACCT GAGCCCCCAC TGGAGCAGGA CCAGGGCCCG
      T  G  E   P  R  P   E  P  P  L   E  Q  D   Q  G  P

151 GGAGAGCGGG GGGCCAGCGG GGGGCTGGCC ACCCTGTGCA ACTTTTGCAA
    G  E  R  G   A  S  G  G  L  A   T  L  C  N   F  C  K

201 ACACAATGGG GAATCTCGCC ACGTGTACTC CTCGCACCAG CTGAAGACAC
     H  N  G   E  S  R  H   V  Y  S   S  H  Q   L  K  T  P

251 CGGAGGGCGT GGTGGTGTGT CCCATCCTAC GACACTATGT GTGTCCCCTG
      E  G  V   V  V  C   P  I  L  R   H  Y  V   C  P  L

301 TGCGGGGCCA CCGGTGACCA GGCTCACACA CTCAAGTACT GCCCGCTCAA
    C  G  A  T   G  D  Q  A  H  T   L  K  Y  C   P  L  N

351 CGGCGGCCAG CAGTCTCTCT ATCGCCGCAG TGGGCGCAAT TCAGCCGGCC
     G  G  Q   Q  S  L  Y   R  R  S   G  R  N   S  A  G  R

401 GCAAGGTCAA GCGCTGA
      K  V  K   R  *
```

Supplementary Figure S2. Genotypes of the *NANOS2* edited piglets from three litters.

1st litter Piglets from *In vivo* fertilized embryos

#1 piglet (Male)- Tag # 136; Mosaic 3 alleles

```
Nanos WT    CTACTTCAACCTGAGCCAGGTGGTGTTGGGACTGATCCAGAA
Allele 1    CTACTTCAACCTGAGCCAGGT-----TGGGACTGATCCAGAA
Allele 2    CTACTTCAACCTGAGCCAGGTG---TTGGGACTGATCCAGAA
Allele 3    CTACTTCAACCTGAGCCAGGGAC---TGGGACTGATCCAGAA
```

#2 piglet (Male)- Tag #137; Mosaic 3 alleles

```
Nanos WT    CTACTTCAACCTGAGCCAGGTGG-TGTTGGGACTGATCCAGAA
Allele 1    CTACTTCAACCTGAGCCAGGTGGGTGTTGGGACTGATCCAGAA
Allele 2    CTACTTCAACCTGAGCCAGGTG-TGTTGGGACTGATCCAGAA
Allele 3    CTACTTCAACCTGAGCCAGGTG---TTGGGACTGATCCAGAA
```

#3 piglet (Female)- Tag #138; Heterozygous KO

```
Nanos WT    CTACTTCAACCTGAGCCAGGTGGTGTTGGGACTGATCCAGAA
Allele 1    CTACTTCAACCTGAGCCAGGT-----TGGGACCGATCCAGAA
Allele 2    CTACTTCAACCTGAGCCAGGTGGTGTTGGGACTGATCCAGAA
```

#4 piglet (Female)- Tag #140; Heterozygous KO

```
Nanos WT    CTACTTCAACCTGAGCCAGGTGGTGTTGGGACTGATCCAGAA
Allele 1    CTACTTCAACCTGAGCCAGGTG---TTGGGACTGATCCAGAA
Allele 2    CTACTTCAACCTGAGCCAGG----------ACTGATCCAGAA
```

2nd litter Piglets from *In vivo* fertilized embryos

#1 piglet (Female) - Tag #141; Heterozygous KO

```
Nanos WT    CTACTTCAACCTGAGCCAGGT-G--GTGTTGGGACTGATCCAGAA
Allele 1    CTACTTCAACCTGAGTCAGGT------GTTGGGACTGATCCAGAA
Allele 2    CTACTTCAACCTAAGCCAGGTTGAAGTGTTGGGACTGATCCAGAA
```

#2 piglet (Male) - Tag # 142; Mosaic 3 alleles

```
Nanos WT    CTACTTCAACCTGAGCCAGGTGGTGTTGGGACTGATCCAGAA
Allele 1    CTACTTCAACCTGAGCCAGGTGT---TGGGACTGATCCAGAA
Allele 2    CTACTTCAACCTGAGCCAGGT-----TGGGACTGATCCAGAA
Allele 3    ---200bp Deletion-------GTTGGGACTGATCCAGAA
```

#3 piglet (Male) - Tag # 143; Mosaic 3 alleles

```
Nanos WT    CTACTTCAACCTGAGCCAGGTGGTGTTGGGACTGATCCAGAA
Allele 1    CTACTTCAACCTGAGCCAGG----------ACTGATCCAGAA
Allele 2    CTACTTCAACCTGAGCCAGGTG---TTGGGACTGATCCAGAA
Allele 3    CTACTTCAACCTGAGCCAGGT-----TGGGACTGATCCAGAA
```

3rd litter Piglets generated from *In vitro* fertilized embryos

#1 piglet (Female) - Tag #145; Heterozygous KO

```
Nanos WT    CTACTTCAACCTGAGCCAGGTGGTGTTGGGACTGATCCAGAA
Allele 1    CTACTTCAACCTGAGCCAGGTG---TTGGGACTGATCCAGAA
Allele 2    CTACTTCAACCTGAGCCAGGTG-TGTTGGGACTGATCCAGAA
```

#2 piglet (Female) - Tag #150; Homozygous KO

```
Nanos WT    CTACTTCAACCTGAGCCAGGTGGTGTTGGGACTGATCCAGAA
Allele 1    CTACCTCAACCTGAGCCAGG-GGTG---GGACTGATCCAGAA
Allele 2    CTACTTCAACCTGAGCCTGGT-GTGTTGGGACTGATCCAGAA
```

#3 piglet (Female) - Tag #149; Biallelic in-frame

```
Nanos WT    CTACTTCAACCTGAGCCAGGTGGTGTTGGGACTGATCCAGAA
Allele 1    CTACTTCAACCTGAGCCAGGTG---TTGGGACTGATCCAGAA
Allele 2    CTACTTCAACCTGAGCCAGGTG---TTGGGACTGATCCAGAA
```

#4 piglet (Male) - Tag #146; Homozygous KO

```
Nanos WT    CTACTTCAACCTGAGCCAGGTGGTGTTGGGACTGATCCAGAA
Allele 1    CTACTTCAACCTGAGCCAGGTC----TGGGACTGATCCAGAA
Allele 2    ---150bp Deletion---------TGGGACTGATCCAGAA
```

#5 piglet (Male)-Euthanized; Heterozygous KO

```
Nanos WT    CTACTTCAACCTGAGCCAGGTGGTGTTGGGACTGATCCAGAA
Allele 1    CTACTTCAACCTGAGCCAGGTTGAAGTGTTGGGACTGATCCAGAA
Allele 2    CTACTTCAACCTGAGCCAGGT------GTTGGGACTGATCCAGAA
```

#6 piglet (Male)- Tag #148; Biallelic in-frame

```
Nanos WT    CTACTTCAACCTGAGCCAGGTGGTGTTGGGACTGATCCAGAA
Allele 1    CTACTTCAACCTGAGCCAGGTGT---TGGGACTGATCCAGAA
Allele 2    CTACTTCAACCTGAGCCAGGTGT---TGGGACTGATCCAGAA
```

#7 piglet (Male)- Euthanized; Homozygous KO

```
Nanos WT    CTACTTCAACCTGAGCCAGGTGGTGTTGGGACTGATCCAGAA
Allele 1    CTACTTCAACCTGAGCCAGGT-----TGGGACTGATCCAGAA
Allele 2    CTACTTCAACCTGAGCCAGGT-----TGGGACTGATCCAGAA
```

#8 piglet (Male) - Tag #144; Homozygous KO

```
Nanos WT    CTACTTCAACCTGAGCCAGGTGGTGTTGGGACTGATCCAGAA
Allele 1    CTACTTCAACCTGAGCCAGGTG-TGTTGGGACTGATCAATAA
Allele 2    CTACTTCAACCTGAGCCAGTGT----TGGGACTGATCCAGAA
```

#9 piglet (Male)- Euthanized; Heterozygous KO

```
Nanos WT    CTACTTCAACCTGAGCCAGGTGGTGTTGGGACTGATCCAGAA
Allele 1    CTACTTCAACCTGAGCCAGGTG---TTGGGACTGATCCAGAA
Allele 2    CTACTTCAACCTGAGCCAGGCGGGGTTGGGACTGATCCAGAA
```

#10 piglet (Male)- Tag #147; Heterozygous KO

```
Nanos WT    CTACTTCAACCTGAGCCAGGTGGTGTTGGGACTGATCCAGAA
Allele 1    CTACTTCAACCTGAGCCAGGTGTT---GGGACTGATCCAGAA
Allele 2    CTACTTCAACCTGAGCCAGGT--T---GGGACTGATCCAGAA
```

#11 piglet (Male) - Tag #251; Mosaic 3 alleles

```
Nanos WT    CTACTTCAACCTGAGCCAGGTGGTGTTGGGACTGATCCAGAA
Allele 1    CTACTTCAACCTGAGCCAGGTG-TGTTGGGACTGATCCAGAA
Allele 2    CTACTTCAACCTGAGCCAG---------------------AA
Allele 3    CTACTTCAACCTGAGCCAGGTGG---TGGGACTGATCCAGAA
```

Supplementary Figure S3. Cross-section of testicular tissue from homozygous *NANOS2* knockout boar 144 at 4 months of age. Note intact seminiferous tubules but complete absence of germline in all tubules.

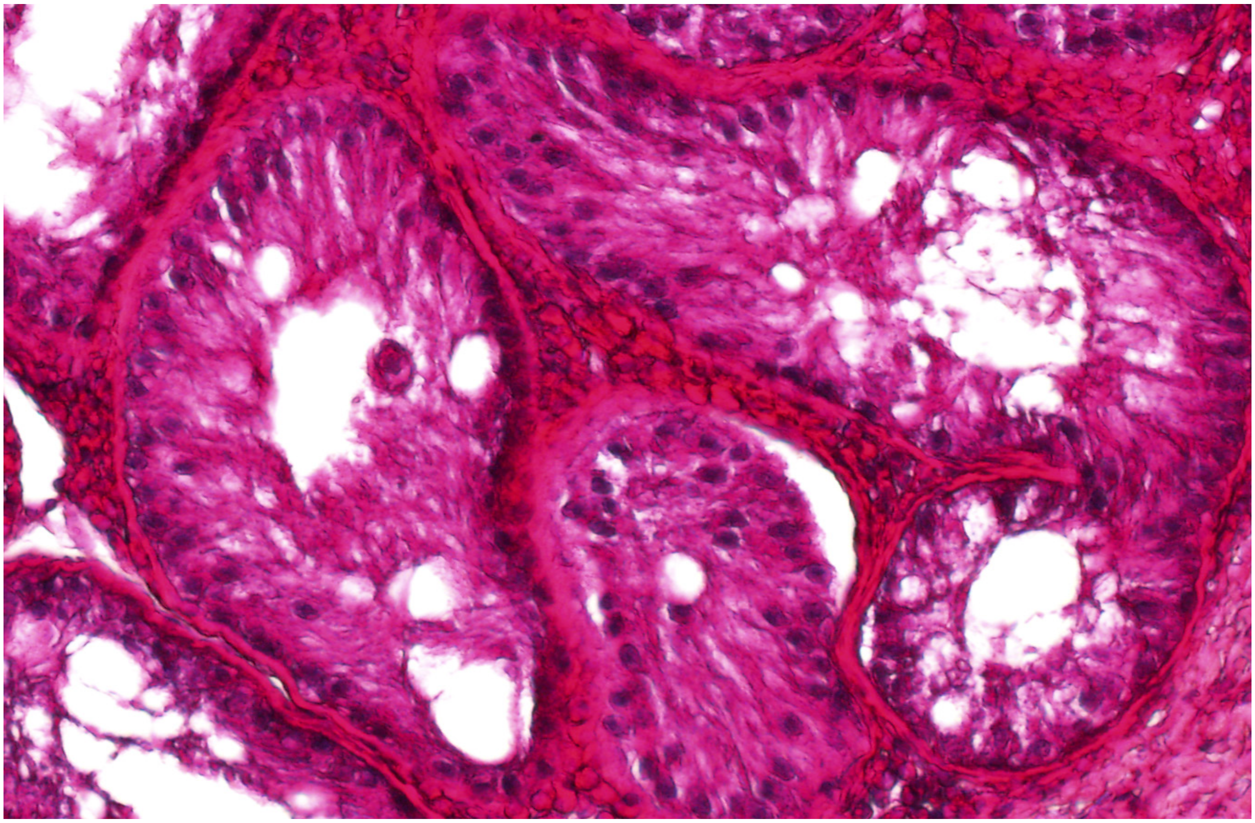

Supplementary Table S1. Summary of genotypes for *NANOS2* mutant gilts.

Gilt #	Ear biopsy genotyping	Genotype classification
138	5bpΔ/WT	Heterozygous KO
140	3bpΔ/10bpΔ	Heterozygous KO
141	3bp ins/3bpΔ	Bi-allelic in-frame
145	1bpΔ/3bpΔ/21bpΔ	Mosaic
149	3bpΔ/4bpΔ	Heterozygous KO
150	3bpΔ/3bpΔ	Bi-allelic in-frame

Supplementary Table S2. Summary of genotypes of *NANOS2* mutant F1 piglets.

Litter 1: Breeding pair (#142 X #145)			
Piglet #	Gender	Ear biopsy genotyping	Genotype classification
101	Male	21 bpΔ / 176 bpΔ	Heterozygous KO
102	Male	1 bpΔ / 3 bpΔ	Heterozygous KO
103	Male	3 bpΔ / 21 bpΔ	Heterozygous KO
104	Male	21 bpΔ / 176 bpΔ	Heterozygous KO
105	Male	1 bpΔ / 1 bpΔ	Homozygous KO
106	Male	1 bpΔ / 3 bpΔ	Heterozygous KO
107	Female	21 bpΔ / 176 bpΔ	Heterozygous KO
108	Female	1 bpΔ / 1 bpΔ	Homozygous KO
109	Female	1 bpΔ / 1 bpΔ	Homozygous KO
110	Female	1 bpΔ / 3 bpΔ	Heterozygous KO

Litter 2: Breeding pair (#142 X #149)			
Piglet #	Gender	Ear biopsy genotyping	Genotype classification
121	Male	3 bpΔ / 3 bpΔ	Biallelic in-frame
122	Male	1 bpΔ / 3 bpΔ	Heterozygous KO
123	Male	1 bpΔ / 3 bpΔ	Heterozygous KO
124	Male	1 bpΔ / 3 bpΔ	Heterozygous KO
125	Male	3 bpΔ / 4 bpΔ	Heterozygous KO
126	Male	3 bpΔ / 4 bpΔ	Heterozygous KO
127	Male	1 bpΔ / 3 bpΔ	Heterozygous KO
128	Female	3 bpΔ / 4 bpΔ	Heterozygous KO
129	Female	1 bpΔ / 3 bpΔ	Heterozygous KO
130	Female	1 bpΔ / 3 bpΔ	Heterozygous KO
131	Female	3 bpΔ / 4 bpΔ	Heterozygous KO
132	Female	3 bpΔ / 4 bpΔ	Heterozygous KO
133	Female	1 bpΔ / 3 bpΔ	Heterozygous KO
Dead1	Male	3 bpΔ / 3 bpΔ	Biallelic in-frame
Dead2	Male	1 bpΔ / 3 bpΔ	Heterozygous KO
Dead3	Female	3 bpΔ / 4 bpΔ	Heterozygous KO

Litter#3: Breeding pair (#140 X #143)			
Piglet #	Gender	Ear biopsy genotyping	Genotype classification
126	male	10bpΔ/10bpΔ	Homozygous KO
127	male	3bpΔ/3bpΔ	Biallelic in-frame
128	male	3bpΔ/3bpΔ	Biallelic in-frame
129	female	10bpΔ/10bpΔ	Homozygous KO
130	female	3bpΔ/3bpΔ	Biallelic in-frame
131	female	3bpΔ/3bpΔ	Biallelic in-frame
**Stillborn**	male	3bpΔ/3bpΔ	Biallelic in-frame

Supplementary Figure S4. Summary of genotypes of *clonal **#146*** piglets

Amplification of *NANOS2* KO NT piglets genomic DNA (#146 boar SCNT piglets)

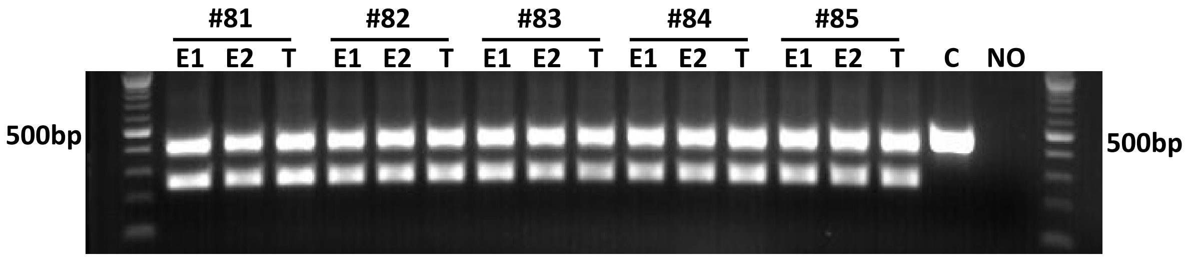

E1: 1st ear E2: 2nd ear T: tail C: control NO: no genomic DNA

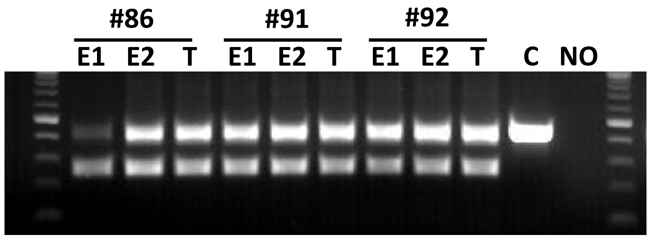

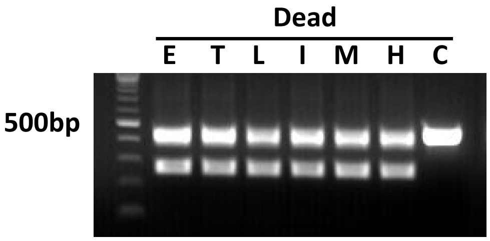

E: ear T: tail L: liver I: intestine
M: mussel H: heart C: control
